# MST4 Kinase Inhibitor Hesperadin Attenuates Autophagy and Behavioral Disorder via the MST4/AKT Pathway in Intracerebral Hemorrhage Mice

**DOI:** 10.1155/2020/2476861

**Published:** 2020-02-03

**Authors:** Xiaodong Wu, Jinting Wu, Wenjie Hu, Qinghua Wang, Hairong Liu, Zhaohu Chu, Kun Lv, Yang Xu

**Affiliations:** ^1^Key Laboratory of Noncoding RNA Transformation Research of Anhui Higher Education Institution (Wannan Medical College), The First Affiliated Hospital of Wannan Medical College, Wuhu, 241000 Anhui, China; ^2^Department of Neurology, The Second Affiliated Hospital of Wannan Medical College, Wuhu, 241000 Anhui, China; ^3^The Second Affiliated Hospital of Wannan Medical College, Wuhu, 241000 Anhui, China; ^4^Department of Neurology, The First Affiliated Hospital of Wannan Medical College, Wuhu, 241000 Anhui, China; ^5^Department of Public Administration, Wannan Medical College, Wuhu, 241000 Anhui, China

## Abstract

**Background:**

The aim of this study was to explore the role of hesperadin in intracerebral hemorrhage (ICH) mice, with the involvement of the mammalian ste20-like kinase 4 (MST4)/AKT signaling pathway.

**Methods:**

All mice were divided into four groups: sham group, sham+hesperidin group, ICH group, and ICH+hesperadin group. The effects of hesperadin were assessed on the basis of brain edema and neurobehavioral function. Furthermore, we observed MST4, AKT, phosphorylation of AKT (pAKT), and microtubule-associated protein light chain 3 (LC3) by western blotting. Protein localization of MST4 and LC3 was determined by immunofluorescence.

**Results:**

The expression of MST4 was upregulated at 12 h and 24 h after ICH. Brain edema was significantly decreased and neurological function was improved in the hesperadin treatment group compared to the ICH group (*P* < 0.05). Hesperadin decreases the expressions of MST and increases pAKT after ICH. Autophagy significantly increased in the ICH group, while hesperadin reduced this increase.

**Conclusion:**

Hesperadin provides neuroprotection against ICH by inhibiting the MST4/AKT signaling pathway.

## 1. Introduction

Intracerebral hemorrhage (ICH) as a subtype of stroke is associated with severe neurological deficit and high mortality [1–3]. Currently, there is still a lack of effective treatment for brain injury after ICH. Autophagy, as a lysosomal degradation pathway, is the main cellular process of cytoplasmic organelle degradation and longevity, misfolding, or damage of proteins [4]. As an important cell death mechanism, autophagy is concerned with neurons after ICH [5–7].

Mammalian ste20-like kinase 4 (MST4), a member of the GCKIII family of kinases, is highly expressed in the brain, placenta, thymus, and peripheral blood leukocytes [8, 9]. The three members of the subfamily include STK25, MST3, and MST4 [10]. MST4 is composed of 416 amino acids with a molecular weight of 46 kDa located on chromosome Xq26 [9]. Xiong et al. reported that the MST4 kinase regulates the proliferation and survival of pituitary cells through the p38 MAPK and AKT signaling cascades [11]. AKT, a multiform serine/threonine kinase that plays a key role in promoting cell survival, was reported to be a downstream target of MST4 [12]. Huang et al. suggested that MST4 mediates the expression of LC3 in the autophagy pathway [13]. The presence of microtubule-associated protein light chain 3 (LC3) in autophagosomes and the transformation of LC3-II are markers of autophagy [14]. Hesperadin was reported to be an ATP competitive inhibitor of Aurora B kinase, inhibiting cell proliferation by decreasing Aurora B activity [15]. HeLa cells treated by hesperadin indicated defects of alignment and separation, whereas sister chromatid separation was complete [16]. Hesperadin inhibits the clinical isolation of various influenza a and b viruses [17]. Recently, Xiong et al. indicated that hesperadin is an effective and selective inhibitor of MST4 kinase [12]. With the increasing exploration of brain injury mechanisms after ICH, prevention of brain injury and the promotion of neuronal survival have become the treatment targets. The present study is aimed at exploring whether the MST4-specific inhibitor hesperadin improves neurological function by inhibiting the MST4-mediated autophagy pathway.

## 2. Materials and Methods

### 2.1. Animals

Male C57BL/6 mice (7-8 weeks old) were purchased from Qinglong Mountain Farm (Nanjing, China). All mice were kept in separate cages and given free access to standard laboratory feed and water. All procedures used in this study conformed to the NIH guidelines for the Care and Use of Laboratory Animals and were approved by the Ethics Committee for the Use of Experimental Animals at Wannan Medical College.

### 2.2. ICH Model

Induction of an intracerebral hemorrhage model by injection of collagenase IV has been described previously [18]. Mice were anesthetized by intraperitoneal injection of 400 mg/kg chloral hydrate and positioned in a stereo positioner (Yuyan type YAN-1 Instrument, Shanghai, China). Experimental ICH was induced into basal ganglia by sterically directed injection of type IV collagenase (0.075 units in 500 *μ*l PBS). The position of the basal ganglia was 2.0 mm to the right of the midline, 0.8 mm anterior to the bregma, and 3.5 mm ventral to the cortical surface. The microsampler was injected for more than five minutes, and the needle is left for another 5 minutes. Bone wax was used to seal the burrs and close the wound. Sham mice were injected with 50 *μ*l 0.9% sterile saline in the same way, instead of autogenous blood. Mice were maintained at 37 ± 0.5°C by a heating lamp. Mice in the control group were untreated. Sham-operated mice went through the same procedure, with sterile physiological saline instead of type IV collagenase.

### 2.3. Experimental Procedure

**Figure 1:**
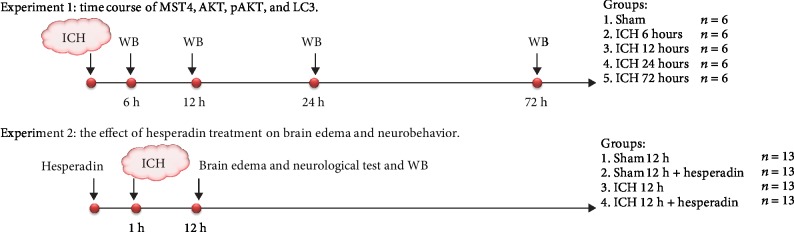
Time-line of study design. Representative figure showing experimental design and number of animals for each group. ICH: intracerebral hemorrhage; WB: western blot.

The experiments were conducted as follows (Figure 1). In this study, mice were randomly assigned to the following two separate experiments. All experiments were performed by two experimenters who were blind to the experimental design.

#### 2.3.1. Experiment 1

To define the time course of MST4, pAKT, AKT, and LC3 after ICH, mice were randomly divided into five groups: sham, ICH 6 h, ICH 12 h, ICH 24 h, and ICH 72 h (*n* = 6). We compared the expression of MST4, AKT, pAKT, and LC3 in different groups by western blot (*n* = 6) and neurological evaluation including the Garcia test and the corner test (*n* = 6). A total of 30 mice were used for experiment 1.

#### 2.3.2. Experiment 2

In order to detect whether MST4 activity was involved in the AKT- and LC3-related autophagy pathway and whether MST4 had an effect on cognition after intracerebral hemorrhage in mice, we compared mice treated with hesperadin (MedChemExpress, HY-12054, USA) with nontreated mice. Hesperadin was injected intraventricularly 1 h before ICH. According to the results of experiment 1, 12 hours after cerebral hemorrhage was determined as the time point. Mice were randomly divided into four groups: sham group, sham+hesperadin group, ICH group, and ICH+hesperadin group (*n* = 13 in each group). In each group, mice were selected randomly for western blot (*n* = 6), immunofluorescence (*n* = 3), and brain edema (*n* = 4). A total of 52 mice were used for experiment 2, and all mice in the four groups were examined for behavior before euthanasia.

### 2.4. Drug Administration

Hesperadin (MCE, HY-12054, 0.5 *μ*g/*μ*l) was dissolved in 1% DMSO and given via intracerebroventricular (i.c.v.) injection 1 hour before ICH (2 *μ*l per mouse). A small hole was formed at the left side of the anterior fontanelle at 1.0 mm, and a no. 27 Hamilton needle was lowered to a depth of 2.3 mm and hesperadin was injected at 0.67 *μ*l/min.

### 2.5. Garcia Test

The Garcia Neuroscore evaluated animal sensory motor performance through seven tests, including (1) spontaneous movement, (2) side stroking, (3) vibrating proprioception, (4) limb symmetry, (5) lateral rotation, (6) forelimb walking, and (7) climbing. Results were collected 12 hours later in a blind way, as previously described [19].

### 2.6. Corner Turn Test

Mice were allowed to enter a corner at an angle of 30 degrees. The direction of the mice turning is recorded, including right or left when mice exit the corner. This was repeated 10 times for at least 30 seconds in the test room, calculating the right turn time. Only fully reared turns involving either wall are included (i.e., ventral folds or horizontal turns excluded).

### 2.7. Brain Edema

To determine brain edema, mice were sacrificed after deep anesthesia and beheaded at 12 h after ICH. The brain was divided into five parts, including the contralateral cortex (cont-cx), the basal ganglia (cont-bg), the ipsilateral cortex (ipsi-cx), the basal ganglia (ipsi-bg), and the cerebellum. The dry and wet weight method was used to calculate brain edema.

### 2.8. Western Blot Analysis

Mice were decapitated at the time of each experimental procedure, and the right cerebral hemisphere was separated and homogenized in RIPA buffer (Servicebio, Wuhan, China). Western blotting was performed as previously described [20]. The primary antibodies included anti-MST4 (1 : 1000, Proteintech), anti-AKT (1 : 1000, Cell Signaling Technology), anti-pAKT (1 : 1000, Cell Signaling Technology), anti-LC3A/B (1 : 1000, Cell Signaling Technology), and *β*-actin (1 : 1000, Cell Signaling Technology).

### 2.9. Immunofluorescence

Immunofluorescence for the brain was conducted on paraffin sections as previously described [20]. At 12 hours after ICH, deeply anesthetized mice were perfused with cold PBS and then infused with 4% paraformaldehyde. The brains were fixed in formalin at 4°C overnight after removal and then dehydrated with 30% sucrose in PBS. Coronal brain slices were sectioned in a cryostat and subjected to immunofluorescence analysis. After degreasing and rehydration, the coronal sections were incubated in EDTA antigen regeneration buffer, rinsed with 5% normal goat serum, and blocked. In a single immunofluorescence labeling section, the coronal sections were then incubated with primary antibodies at 4°C overnight, including rabbit anti-MST4 (1 : 100, Proteintech) and rabbit anti-LC3A/B (1 : 100, Cell Signaling Technology), then with anti-rabbit IgG : CY3 and anti-rabbit IgG : FITC, respectively. In a double immunofluorescence labeling section, blocked sections were incubated with LC3A/B antibody overnight and then with HRP-labeled secondary antibodies. After rinsing, sections were incubated in anti-rabbit IgG: CY3 for 10 minutes and then with EDTA antigen regeneration buffer. The same sections were then incubated with MST4 antibody overnight and then with HRP-labeled secondary antibodies. All the sections were finally exposed to DAPI (Servicebio, Wuhan, China) to display nuclear changes.

### 2.10. Statistical Analysis

All data were expressed as mean ± SD. GraphPad Prism 6 was used for statistical analysis. One-way ANOVA was used to analyze the results from different groups, and the significance was indicated by *P* value < 0.05.

## 3. Results

### 3.1. The Time Course of MST4, AKT, pAKT, and LC3 Expression and Neurological Function in ICH Mice

**Figure 2:**
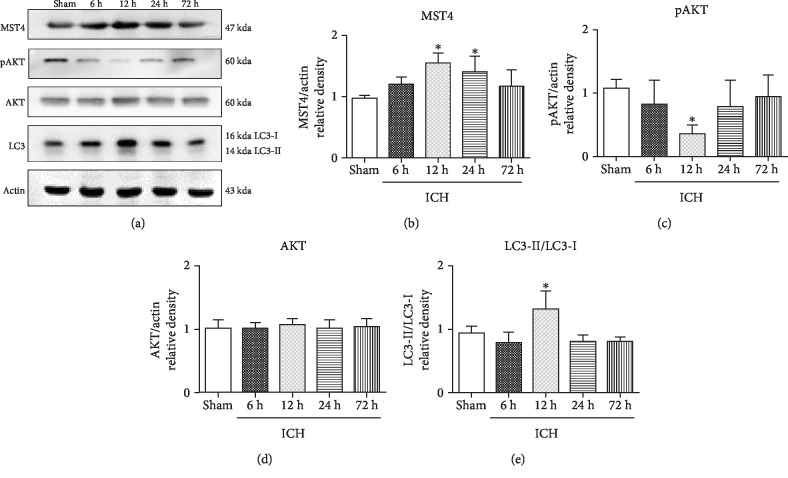
Endogenous expression of MST4, pAKT, AKT, and LC3 after ICH. (a) Representative western blot bands for MST4, pAKT, AKT, and LC3 expression in sham and ICH mice 6, 12, 24, and 72 h following ICH. Densitometric quantification of (b) MTS4/actin, (c) pAKT/actin, (d) AKT/actin, and (e) LC3-II/LC3-I for western blot. Data were expressed as mean ± SD. ^∗^*P* < 0.05 versus sham; *n* = 6 animals for each group.

**Figure 3:**
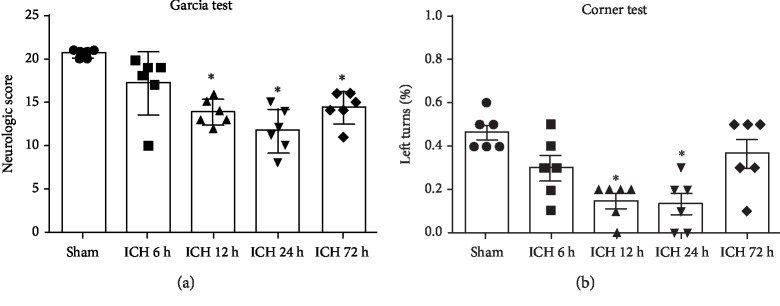
Neurological function evaluation after ICH. (a) Garcia test and (b) corner turn test at 6, 12, 24, and 72 h following ICH. Data were expressed as mean ± SD. ^∗^*P* < 0.05 versus sham; *n* = 6 animals for each group.

We determined the temporal expression of the MST4 protein and the autophagy-associated protein including AKT, pAKT, and LC3 at different times following ICH (Figure 2(a)). MST4 expression was significantly peaked at 12 h following ICH (Figure 2(b), *P* < 0.05). There was no significant change for the AKT expression, while pAKT expression decreased (Figures 2(c) and 2(d), *P* < 0.05). Autophagy marker LC3 was increasingly expressed at 12 h following ICH (Figure 2(e), *P* < 0.05). The results of the neurological score showed that neurological function deteriorated at 12 h, 24 h, and 72 h after ICH (Figures 3(a) and 3(b), *P* < 0.05).

### 3.2. Hesperadin Reversed ICH-Induced Autophagic Activation

**Figure 4:**
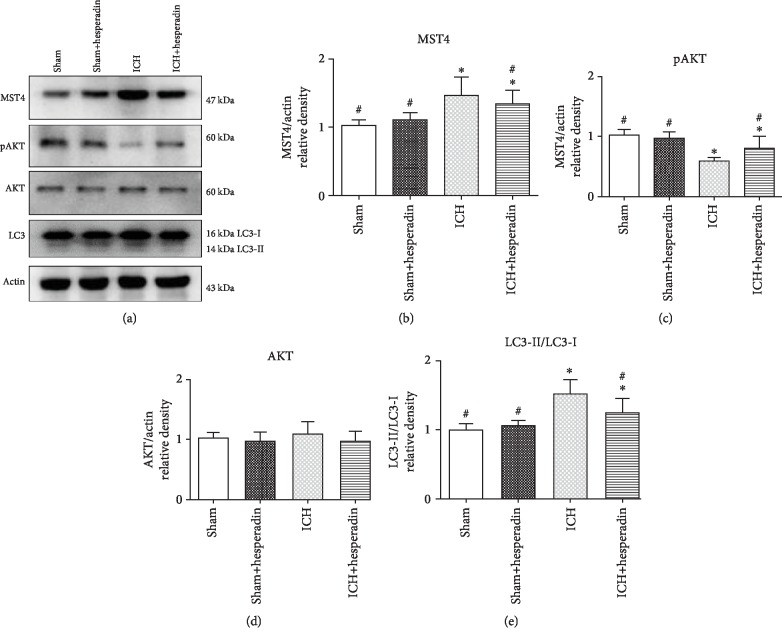
Administration of hesperadin influenced endogenous expression of MST4, pAKT, AKT, and LC3 12 h following ICH. (a) Representative western blot bands for MST4, pAKT, AKT, and LC3 expression in sham and ICH mice 12 h following ICH. Densitometric quantification of (b) MTS4/actin, (c) pAKT/actin, (d) AKT/actin, and (e) LC3-II/LC3-I for western blot. Data were expressed as mean ± SD. ^∗^*P* < 0.05 versus sham; ^#^*P* < 0.05 versus vehicle; *n* = 6 animals for each group.

**Figure 5:**
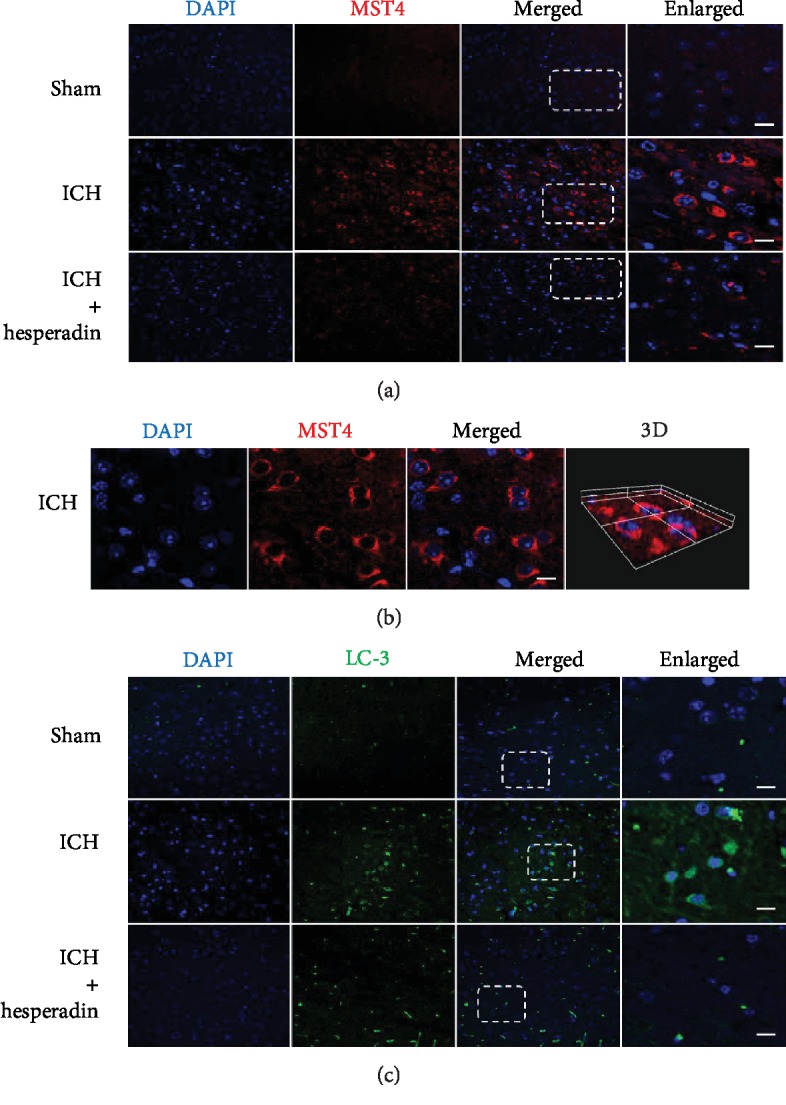
Immunofluorescence staining of MST4 and LC3 at 12 h after ICH. Representative images of immunofluorescence staining to show the expression of (a) MST4 (red), (b) stereoscopic 3D version of the local expression, and (c) LC3 (green). Images of brain samples were obtained from the perihematoma area 12 h following ICH. *n* = 3 mice/group. Scale bar: 20 *μ*m.

**Figure 6:**
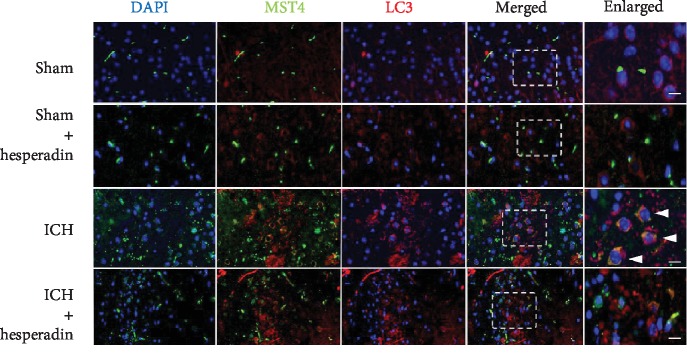
Pretreatment with hesperadin inhibits the colocalization of MST4 and LC3. MST4 (green) and LC3 (red) overlap as shown by triangles. Images of brain samples were obtained from the perihematoma area 12 h following ICH. *n* = 3 mice/group. Scale bar: 20 *μ*m.

According to the results of experiment 1, the time point of cerebral hemorrhage in the ICH group and the hesperadin treatment group was set to 12 hours. MST4, pAKT, AKT, and LC3 showed different changes in experiment 2 (Figure 4(a)). The expression of MST4 was significantly decreased (Figure 4(b), *P* < 0.05). On the contrary, pAKT was increased in the hesperadin treatment group compared to the ICH group (Figure 4(c), *P* < 0.05). AKT showed no significant change (Figure 4(d), *P* < 0.05), while LC3 decreased in the hesperadin group compared to the ICH group (Figure 4(e), *P* < 0.05). The expression of MST4, pAKT, AKT, and LC3 showed no significant difference between the sham group and the sham+hesperidin group (Figure 4, *P* > 0.05). The above results indicated that hesperidin blocks the AKT protein-related pathway autophagy by inhibiting MST4. Immunofluorescence staining showed that hesperadin decreased the ICH-induced upregulation of MST4 and the autophagy marker LC3. MST4 fluorescence was located in the cytoplasm, mainly around the nucleus. In the hesperadin group, MST4 was significantly reduced compared to the ICH group. Similarly, LC3 expression was parallel with MST4 (Figure 5). In the sham group, MST4 and LC3 showed weaker fluorescence intensity and dotted distribution, while they colocalized around the nucleus and in the ICH group. With hesperadin pretreatment, MST4 and LC3 staining were significantly reduced and nonoverlapping (Figure 6).

### 3.3. Hesperadin Reduced Brain Edema and Improved Neurobehavior at 12 Hours after ICH

**Figure 7:**
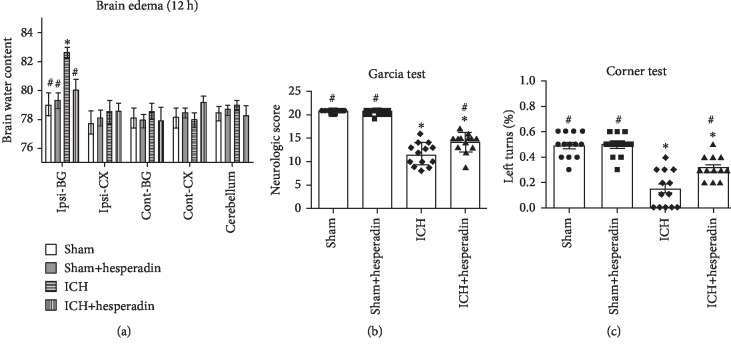
Administration of hesperadin decreased brain edema and improved neurological function 12 h after ICH: (a) brain edema, *n* = 4 mice/group; (b) Garcia test, *n* = 13 mice/group; (c) corner turn test, *n* = 13 mice/group. ^∗^*P* < 0.05 versus sham; ^#^*P* < 0.05 versus vehicle; *n* = 13 animals for each group.

The ICH+hesperadin group showed significant brain edema reduction in the ipsilateral basal ganglia compared to the ICH group; moreover, no significance was found between groups for other parts of the brain (Figure 7(a), *P* < 0.05). Hesperadin treatment in ICH mice significantly improved neurological deficits compared to the control group (Figures 7(b) and 7(c), *P* < 0.05). There was no significant difference in brain water content and behavioral function between the sham group and the sham+hesperidin group (Figure 7, *P* > 0.05).

## 4. Discussion

Despite much research on ICH pathology, effective neuroprotective therapy remains limited [21, 22]. The pathological mechanism associated with ICH is complex and involves different cell signaling pathways, resulting in neuronal death and cell stress injury. It has been reported that autophagy pathway activation was detected in neurons after ICH [6]. Increasing evidence has indicated that the role of autophagy in ICH pathology is crucial [23]. MST4 plays multiple roles and possesses many cell-specific functions. MST4 shows a proapoptotic effect in breast cancer cells [24]. Moreover, MST4 may regulate cell migration in HeLa cells [25]. It has been reported that MST4 participates in autophagy, leading to enhanced autophagic flux [13].

The first objective of this study was to elucidate the changes and effects of MST4 expression in ICH. Despite that MST4 is clearly expressed in the brain, the role of altered expression and pathology is not clear, especially in cardiocerebrovascular diseases. Our study shows that MST4 is extensively expressed in healthy brain, whereas it increases and reaches a peak at 12 h following ICH. AKT did not change significantly after ICH, whereas pAKT expression decreased and reached a bottom at 6 h and 72 h following ICH. The expression of the autophagy marker LC3 peaked at 12 h after ICH. Therefore, it indicated that ICH could directly activate autophagy and upregulate MST4 expression. Accordingly, the time point of 12 h after ICH was used for subsequent experiments with a corresponding mechanism. As a consequence of ICH, mice showed different levels of functional impairment. Additionally, pAKT downregulation may be due to the feedback response of MST4 via affecting the phosphorylation of AKT. These observations indicated MST4 may be involved in controlling autophagy in ICH mice.

Our second objective was to investigate whether the novel MST4 inhibitor hesperadin, which was previously considered as an Aurora kinase inhibitor, could effectively inhibit the expression of MST4. Hemorrhage in the basal ganglia is known to cause brain edema and neurological deficits [26]. We confirmed that hesperadin administration for ICH mice significantly improved edema and alleviated neurological deficits at 12 h after ICH compared to the ICH group. MST4 expression in ICH mice treated with hesperadin was significantly lower than ICH mice, which proved the potential of hesperadin as an MST4 inhibitor. We found that hesperadin is neuroprotective, which could ameliorate brain edema and behavioral deficits after experimental ICH in mice. In this study, hesperadin was confirmed to show a neuroprotective effect and can improve brain edema and neurofunction deficits in ICH mice.

Finally, we investigated the regulative role of MST4 on autophagy and its significance in neuronal injury. To further prove our working hypothesis, we inhibit MST4 via the administration of hesperadin. It has been indicated that MST4 suppression would decrease AKT phosphorylation, thereby reducing the formation of autophagosome LC3-II. With the activation of AKT/LC3 during the autophagy process, brain edema and neurological deficits are correspondingly improved. Western blot analysis for MST4 at 12 hours after ICH showed a significant increase compared to the sham group; moreover, it was reduced in the hesperadin-treated group, which confirmed our hypothesis that hesperadin suppressed autophagy caused by ICH. We have detected that MST4 expression was parallel relevant with LC3, and the possible explanation is that downregulation of MST4 surrounding hemorrhage may protect cells by promoting AKT phosphorylation and then inhibiting autophagosome formation. The relationship between MST4 and LC3 has not been reported yet, and there may be a potential relationship between MST4 and autophagy. Our experiments prove the potential relationship between MST4 and autophagy: the expression of MST4 affects the occurrence of autophagy. And this will bring a new perspective to the exploration of autophagy-related pathways in the following exploration.

Based on this study, we concluded that hesperadin may be a neuroprotective factor that alleviates autophagy of nerve cells around hematoma, as well as a significant neurologic improvement. It may provide a new method for exploring the potential molecular and cellular mechanisms between MST4 and AKT after ICH, as well as a special target for the treatment of ICH. However, there are some limitations existing in our research. First, the direct relationship between MST4 and ICH has not been studied and the detailed and exact mechanism by which ICH affects MST4 expression remains to be explored. Second, we only focused on the effect of MST4 on autophagy after ICH. Additionally, our study only tested the recommended dose that was reported and only one time point was selected in the mechanism study. We will further study the mechanism of hesperadin to protect brain injury in ICH.

In conclusion, hesperadin attenuates autophagy via the MST4/AKT pathway in intracerebral hemorrhage mice, and it might provide new views for the treatment of ICH-induced autophagy. Treatment targeting autophagy to limit brain injury or promote recovery after ICH still needs further research.

## Data Availability

The datasets analyzed during the current study are available from the corresponding authors on reasonable request.
